# Comparing data driven and physics inspired models for hopping transport in organic field effect transistors

**DOI:** 10.1038/s41598-021-02737-7

**Published:** 2021-12-08

**Authors:** Madhavkrishnan Lakshminarayanan, Rajdeep Dutta, D. V. Maheswar Repaka, Senthilnath Jayavelu, Wei Lin Leong, Kedar Hippalgaonkar

**Affiliations:** 1 School of Electrical Electronic Engineering, Nanyang Technological University, 50 Nanyang Avenue, Singapore, 639798 Singapore; 2 Institute of Materials Research & Engineering, Agency for Science, Technology and Research (A*STAR), Singapore, 138632 Singapore; 3 Institute for Infocomm Research, Agency for Science, Technology and Research (A*STAR), Singapore, 138632 Singapore; 4 School of Materials Science and Engineering, Nanyang Technological University, 50 Nanyang Avenue, Singapore, 639798 Singapore

**Keywords:** Computational science, Scientific data, Materials for devices, Organic molecules in materials science, Theory and computation, Scaling laws

## Abstract

The past few decades have seen an uptick in the scope and range of device applications of organic semiconductors, such as organic field-effect transistors, organic photovoltaics and light-emitting diodes. Several researchers have studied electrical transport in these materials and proposed physical models to describe charge transport with different material parameters, with most disordered semiconductors exhibiting hopping transport. However, there exists a lack of a consensus among the different models to describe hopping transport accurately and uniformly. In this work, we first evaluate the efficacy of using a purely data-driven approach, i.e., symbolic regression, in unravelling the relationship between the measured field-effect mobility and the controllable inputs of temperature and gate voltage. While the regressor is able to capture the scaled mobility well with mean absolute error (MAE) ~ *O*(10^–2^), better than the traditionally used hopping transport model, it is unable to derive physically interpretable input–output relationships. We then examine a physics-inspired renormalization approach to describe the scaled mobility with respect to a scale-invariant reference temperature. We observe that the renormalization approach offers more generality and interpretability with a MAE of the ~ *O*(10^–1^), still better than the traditionally used hopping model, but less accurate as compared to the symbolic regression approach. Our work shows that physics-based approaches are powerful compared to purely data-driven modelling, providing an intuitive understanding of data with extrapolative ability.

## Introduction

Several classes of organic semiconductors—conjugated polymers and small molecules—have been discovered and explored in a wide range of electronic applications, such as flexible displays^1^, self-healing materials for electronic skin^2^, energy harvesting^3,4^, chemical and biological sensors^5^. The electrical transport of these semiconductors have been studied using time-of-flight (TOF) or charge extraction by linearly increasing voltage (CELIV) measurements^6^ and most commonly by fabricating organic field-effect transistors (OFETs) employed in Hall measurements^7–9^. The electrical transport in these materials is complex and no specific transport mechanism has been established due to the weak interactions between molecules and strong dependence on processing conditions and device morphologies. Such weak van der Waals interactions lead to different degrees of disorder ranging from amorphous to crystalline materials, which affects the electrical transport. Small molecules and single crystals in which π–π interactions dominate possess less degree of disorder and are expected to follow band-like transport^10^. Alternatively, polymers that lack order in packing are thereby expected to follow thermally-driven hopping transport^11^. Thus, thermally activated Arrhenius-type hopping usually describes the electrical transport in conjugated polymers with the assumption that a fraction of the total number of available energy states is occupied. While physical models like the Miller–Abrahams formalism^12^, explaining hopping transport in material systems, are complex involving many parameters that are defined empirically, it provides an intuitive understanding of the physics of transport and hence can be predictive in nature. In essence, this boils down to the dependence of charge hopping upon the thermodynamic temperature (*T*) and the modulation of additional carriers in the polymeric channel via the applied gate voltage (*V*_*g*_) in the OFET configuration.

**Figure 1:**
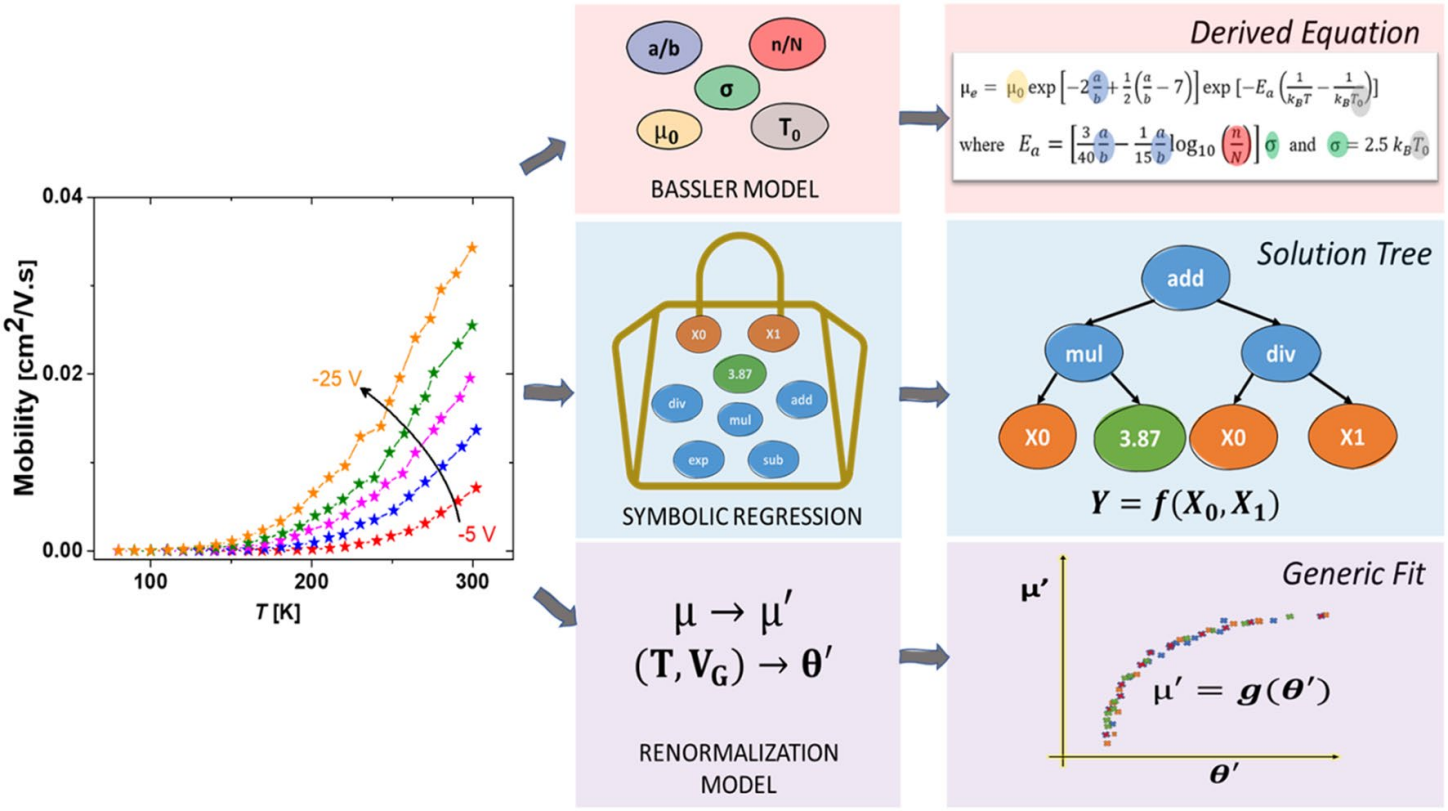
An overview of various approaches to extract the underlying input–output relationships from temperature-dependent field-effect mobility data. Here, the left panel consists of a representative temperature-dependent field-effect mobility data at different gate voltages. The middle panel captures the methods followed in each approach. The panel on the right shows the final solution form for each approach. For the Bassler Model, the unknowns are coloured, and the derived equation fits these values. In Symbolic Regression, inputs (X_0_ and X_1_), constants and mathematical operations are fed into the algorithm, while a solution tree produces a fitting function, Y. For the Renormalization Model, a generic fit by rescaling the inputs is obtained.

Similar efforts at understanding physical principles in other materials and physical systems have been employed in recent times with simulated physics datasets. “AI Feynman” (AI stands for Artificial Intelligence) is a recursive symbolic regression algorithm, which connects neural networks with physics-inspired methods such as dimensionality analysis, generalized symmetries, multiplicative separability, and inversion to discover physical equations that explain the data^13,14^. The use of symbolic neural networks to learn the dynamics of data with a Partial Differential Equation (PDE-NET) fixes the functional form but is able to capture the underlying parameters^15^. Physics-informed Neural Networks (PINNs) respect the laws of physics while solving both differentiable (data-efficient approximators) and discrete datasets and have been used to solve problems in fluids^16^, quantum mechanics and more^17^. In this work, we investigate the efficacy of a powerful mathematical approach, namely symbolic regression (SR), in recapturing underlying physics after fitting transport data of three different OFETs based on organic molecules of p-type pentacene, and n-type buckminsterfullerene (C_60_) as well as conjugated polymer poly(3-hexylthiophene-2,5-diyl) (P3HT). While the regressor can fit the experimental data after carefully tuning the hyperparameters, physical interpretability is not easy and guaranteed in all cases. The regressor does succeed in identifying the physically meaningful and essential terms to describe the transport. However, it is unable to decouple the effect of the activation energy modulated by varying gate voltage from the magnitude of the thermal energy window that is directly proportional to temperature. We see that the regressor treats the whole dataset as one entity, as opposed to a system of five curves following the same bi-parametric equation, where the data in each curve is generated by only varying the value of one of the parameters, while keeping the other parameter constant. The resulting equation fits the entire dataset at once instead of providing a solution that is repeatable curve-by-curve by varying the value of the second variable/parameter (gate voltage in this case). To overcome this missing physical insight, we compare this with a renormalization approach to describe the transport by defining dimensionless quantities from the temperature dependent mobility data. Derivation of dimensionless quantities captures both the effects of temperature as well as gate voltage embedded in a single parameter, rather than in multiple parameters as seen in the existing models^18^, and provides us with a one-shot approach in describing and characterising hopping transport in organic semiconductors. We demonstrate that this approach is powerful as it also helps to differentiate between processing conditions for the same molecules by comparing the extent of activation energy that is accessed over the range of the applied gate voltages. Figure 1 depicts various approaches for extracting input–output relationships involved in hopping transport phenomena.

As a benchmark, we use the model derived by Bassler et al. for the field-effect mobility (*μ*_*e*_) using the effective medium approximation and the concept of effective transport energy^11,19^, while considering Gaussian density of states (DOS) and Miller–Abrahams jump rates for thermally activated Arrhenius-type hopping. Here, according to the so-called Bassler model, ${\mu }_{e}={\mu }_{0}\text{exp}\left[-2\frac{a}{b}+\frac{1}{2}\left(\frac{a}{b}-7\right)\right]\text{exp}\left[-{E}_{a}\left(\frac{1}{{k}_{B}T}-\frac{1}{{k}_{B}{T}_{0}}\right)\right]$, where ${E}_{a}=\left[\frac{3}{40}\frac{a}{b}-\frac{1}{15}\frac{a}{b}{\text{log}}_{10}\left(\frac{n}{N}\right)\right]\upsigma$ and $\upsigma =2.5 {k}_{B}{T}_{0}$.

As illustrated in Fig. S3, *σ* indicates the broadening of the Gaussian DOS, *a/b* is the ratio of inter-site distance and localization radius. Further, *n/N is* the ratio of occupied and total number of transport states. *T*_*0*_ is the isokinetic temperature at which all curves of varying *E*_*a*_ converge; *n is* the number of occupied transport states, which can be tuned by the applied gate voltage (*V*_*g*_) since it is directly proportional to *V*_*g*_. The total number of transport states, *N*, is a constant of the order of ~ 10^23^ cm^−3^, which relates to the lattice constant (*a*) as $N={a}^{-3}$ for an assumed cubic lattice.

In the equation above, the hopping process is affected by both spatial (*a*, *b*) and energetic terms ($${E}_{a})$$. The spatial terms indicate the extent of hopping (given by *a*) and the localization radius between two sites (given by *b*), while the energetic terms specify the activation required for the phonon-assisted excitation, spatial tunnelling, and phonon emission—all together considered as one hopping event—to take place from one site to another. The tunnelling strongly depends on the extent of wave function overlap between neighbouring molecular orbitals. This results in an increase in mobility when the wave functions overlap is higher, which thereby decreases the activation energy for the hopping event^20,21^. The activation energy also relates to the structure and morphology of the semiconducting layer through molecular orientation and processing conditions.

## Data description

**Figure 2:**
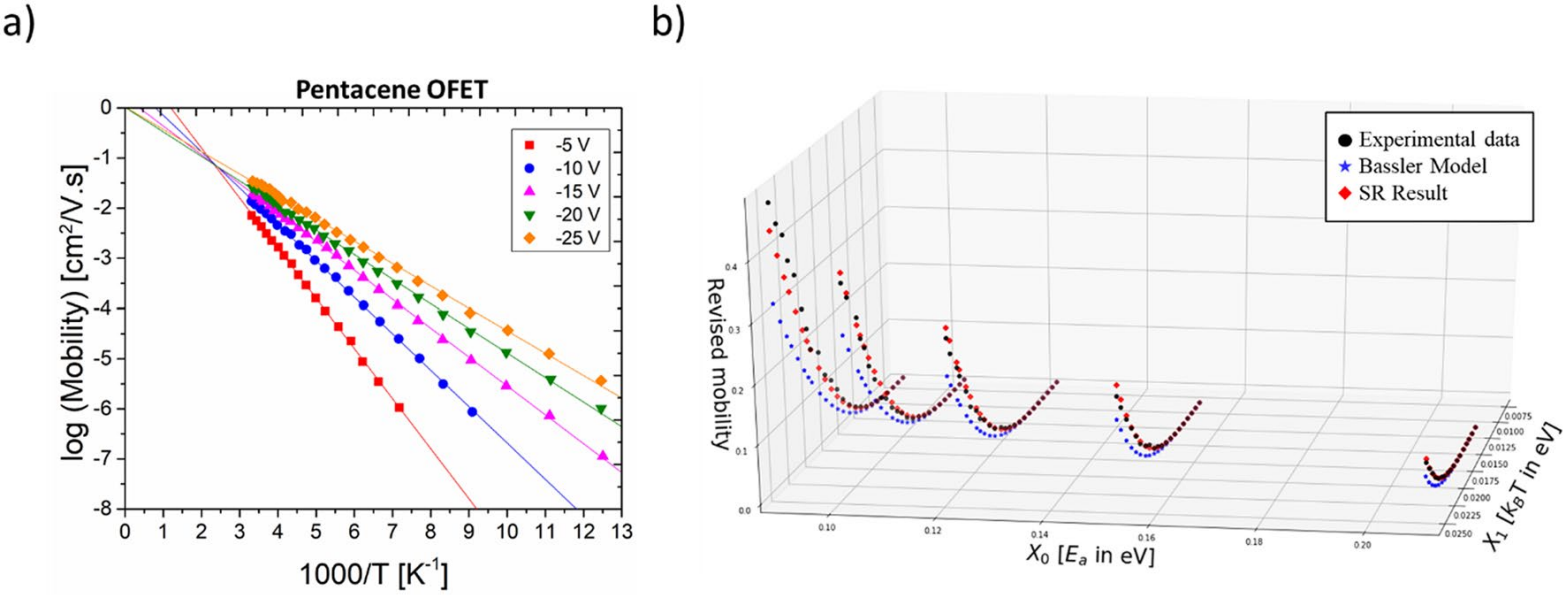
(**a**) Temperature-dependent mobility data for pentacene OFET; (**b**) Simultaneous visualization of the fits obtained from symbolic regression (SR) (red symbol) and the Bassler model (blue symbol) with respect to the experimental data (black symbol) (data extracted from Ref.^19^).

The temperature-dependent mobility data for pentacene, P3HT, C_60_ OFETs is obtained from literature^22–24^. While pentacene and P3HT were fabricated through solution processing methods on Si/SiO_2_ substrates with Au contacts, C_60_ film was fabricated through standard flush evaporation or hot-wall epitaxy^25^ on ITO/divinyltetramethyldisiloxane-bis(benzocyclobutane) (BCB) substrates with LiF/Al top contacts. The pentacene film can be regarded as polycrystalline, while the P3HT film has ordered regions that alternate with disordered regions. The C_60_ film is of greater crystallinity compared to both P3HT and pentacene films. The field-effect mobility (output) data recorded as a function of the change in two variables namely temperature and gate voltage (inputs), is shown in Fig. 2a for pentacene OFET and Fig. S1 for P3HT and C_60_ OFETs. Typical of materials science dataset sizes, each OFET data is comprised of ~ 100 points, stacking input–output information from five temperature-dependent curves at five different gate voltages that were used to record the data (as seen in Fig. 2a). The activation energy can be regulated by varying the applied gate voltage, i.e., a larger gate voltage signifies that a smaller activation energy is needed.

## Symbolic regression: an approach for data-driven learning of OFET behaviour

First, we introduce Symbolic Regression (SR) and discuss the fundamentals of genetic programming and how it is employed specifically in this work to extract the underlying input–output relationship in the form of comprehensive mathematical expressions^26,27^. Although other machine learning methods are useful in extracting input–output relations, they employ predetermined functional forms to fit the data (for instance, polynomial regression). On the contrary, SR attempts to solve such problems without requiring any prior knowledge of the functional forms, by utilizing a finite set of operators and functions (like arithmetic, trigonometric, exponential). Such a symbolic input–output mapping is beneficial as it offers interpretability in applied physics and material science, which is the motivation behind using this technique over other machine learning models^27–29^.

For characterizing hopping-like transport phenomena in OFETs, several models have been proposed to relate the field-effect mobility with inputs like temperature and back-gate voltage. However, these models differ in both the functional forms as well as the nature and number of the transport parameters used. In the present work, SR is applied on each OFET dataset where the input variables are defined as ${X}_{0}= {E}_{a}$ and ${X}_{1}= {k}_{B}T$ and the output variable as $Y\propto \text{log}\left(\frac{{\mu }_{e}}{prefactor}\right)$. The activation energy *E*_*a*_ for each *V*_*g*_ curve is obtained from the slope of the respective curve by expressing the logarithmic mobility as a function of the reciprocal temperature. The thermal energy associated at temperature T is calculated as a product of the temperature T and the Boltzmann constant, *k*_*B*_. This choice of the two input variables, ${X}_{0}= {E}_{a}$ and ${X}_{1}= {k}_{B}T$ is so that both the variables are in the same units (eV) and hence guide the SR outcome expressions into a meaningful and interpretable form, since the range of values for both *E*_*a*_ and *k*_*B*_*T* were similar. This is of special significance because a choice of *E*_*a*_ ≫ *k*_*B*_*T* would mean that the hopping energy is too large, and transport does not take place. On the other hand, *E*_*a*_ ≪ *k*_*B*_*T* would result in a transport mechanism that is different from hopping, akin to metallic conduction, which is unlikely in organic thin films. Further, the usage of dimensionless input variables obtained by normalising the existing X_0_ and X_1_ parameters with the thermal energy at room temperature, i.e., ${k}_{B}(300K)$, was also explored and discarded as it did not help in accuracy or interpretability.

The output variable Y, denoting revised mobility, is a dimensionless quantity. The Bassler model expression can be written in log_10_ scale as,

1 $$\text{log}\left(\frac{{\mu }_{e}}{prefactor}\right)= -\frac{{E}_{a }}{2.303}\left(\frac{1}{{k}_{B}T}-\frac{1}{{k}_{B}{T}_{0}}\right) .$$

where, $\frac{{\mu }_{e}}{prefactor}$ is the dimensionless revised mobility.

The framework applied in this paper seeks to find out a mapping $Y=f({X}_{0},{X}_{1})$. We use the *gplearn* package in Python to perform SR^26^.

GP minimizes the Mean Absolute Error (MAE) between the experimental data and the predictions to produce symbolic expressions. In addition, upon evaluating the best values of SR parameters, specifically, the parsimony coefficient and the tournament size (details can be found in Supplementary Information) are determined using grid search, and the resulting trees render mathematical expressions for the three different OFETs as shown in Table 1. After inspecting the results from SR, we find that the outcome expressions fit the experimental data with an MAE of ~ 0.02–0.03 with a fit better than that offered by the Bassler model; a comparison of the results between the SR outcome and the Bassler Model, is shown in Fig. 2b.

**Table 1:** A comparison of the MAE values obtained from the three different fitting approaches in consideration—symbolic regression (SR), Bassler model and Renormalization fitting—for pentacene, P3HT and C_60_ OFETs.

Molecule	$\text{log}{\mu }_{e}$	MAE from symbolic regression	MAE from Bassler Model	MAE from re-normalization fitting
Pentacene	$-0.52+ 9.37 \left({E}_{a}* {k}_{B}T\right)- 0.38\frac{{E}_{a}}{{k}_{B}T} + 8.99 {E}_{a} - 842.18 {\left({k}_{B}T\right)}^{2} + 46.64 {k}_{B}T$	0.027	0.204	0.224
P3HT	$100 {E}_{a} {k}_{B}T - 0.273\frac{{E}_{a}}{{k}_{B}T} + 3 {E}_{a} + 31.9 {k}_{B}T + 0.138$	0.037	0.145	0.907
C_60_	$-15.31 \left({E}_{a} * {k}_{B}T\right)- 0.47\frac{{E}_{a}}{{k}_{B}T}+ 15.78 {E}_{a} - 9.5 {k}_{B}T + 0.29$	0.0123	0.488	0.0717

The second column consists of the resulting equations obtained from the SR fits.

In Table 1, SR outcome expressions capture the expected terms according to the Bassler model, i.e., *E*_*a*_ and (*E*_*a*_/*k*_*B*_*T)*, and include higher-order non-linear terms. Therefore, the coefficients attached to these terms, expectedly do not match the coefficients in Eq. (1)^27^. In summary, the SR gives us better MAE at a cost of lack of generality from a physical equation, which is what the Bassler Model offers. An innate limitation of the SR approach is limited accuracy with sparse data, which can be mitigated by using more densely populated data^13^.

## Physics-inspired model through renormalization

In order to extract underlying physical equations, we next consider a simple yet robust physics-driven approach that makes use of fewer parameters to characterize the underlying transport phenomena in OFETs. In solid-state physics, the concept of renormalization theory is utilized to renormalize the inputs into dimensionless quantities in such a way that certain critical points are identified as scale-invariant, which captures the physics behind the observed data. This has been employed in the study of second-order magnetic phase transitions, for instance^30,31^.

We consider the same OFET data as used for the SR and the Bassler Model. We notice that the carrier mobility, the average velocity of the charge carrier to the electric field, of these disordered systems strongly depends on the carrier concentration, i.e., on applied gate bias ($\mu \propto {V}_{g}^{n}$, where *n is* the *exponent*)^32^. The carrier mobility of these organic materials is expected to follow a similar trend with several parameters needed to describe its temperature and field-dependent behaviour. Typically, the carrier mobility of disordered systems can be represented with the help of a scaling law given by $\mu \propto {\text{T}}^{\upgamma } {e}^{(\frac{\text{E}}{{\text{k}}_{\text{B}}T})}$^33^.

In this particular case, for each mobility curve at fixed gate bias (*V*_*g*_), *T*_*p*_ is the temperature corresponding to the maximum mobility (*µ*_*p*_) and *T*_*r*_ is the reference temperature corresponding to half of the maximum mobility (*µ*_*p*_*/2*). Due to the limitation of a small number of data points, *T*_*r*_ is obtained by interpolation directly from the mobility-temperature plots. We choose *T*_*p*_ as 300 K as the scale-invariant point (*T*_*p*_) in this work for all the studied OFETs.

**Figure 3:**
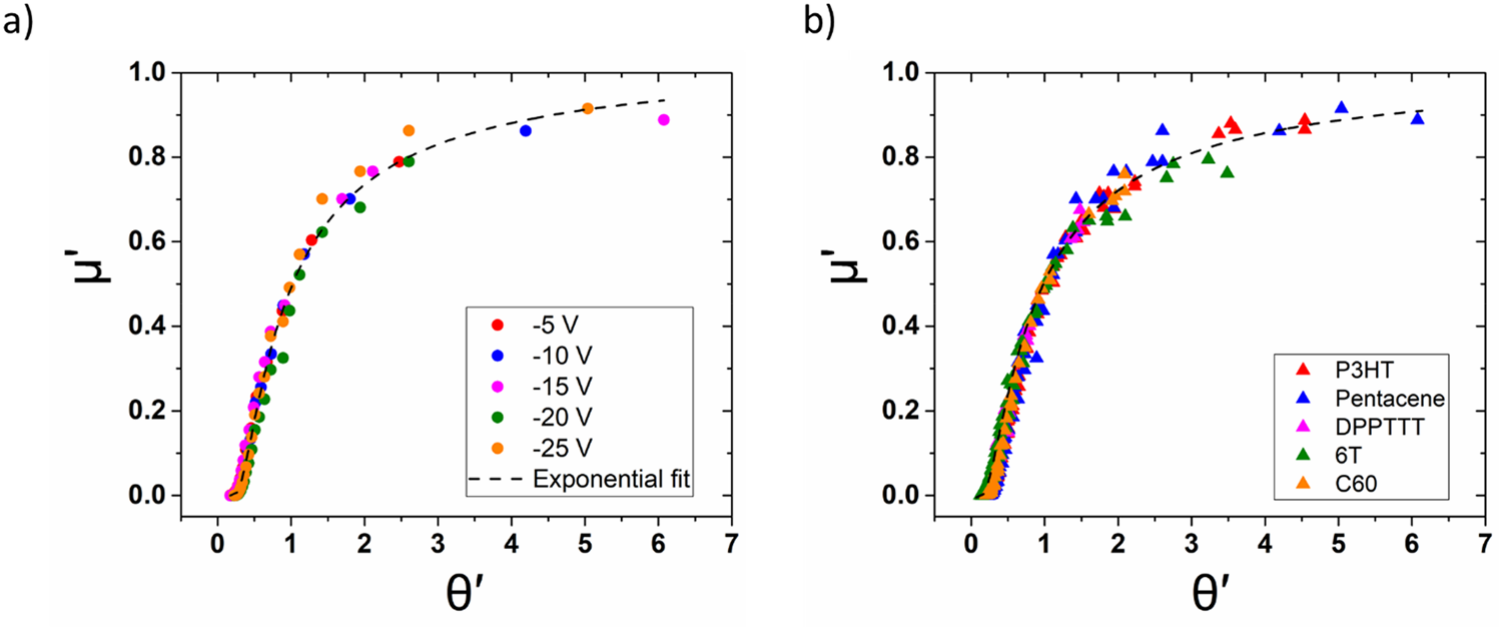
(**a**) Scaled mobility data at different gate voltages (coloured circles) for pentacene OFET and the corresponding exponential curve that fits the scaled data (black dashed line) (data extracted from Ref.^19^); (**b**) juxtaposition of scaled mobility data at different gate voltages for five different molecules (coloured triangles) resulting in all of them following the same exponential function (shown as black dashed line) as observed in (**a**).

Upon extracting the *T*_*r*_ values, we defined two dimensionless quantities ${\mu }^{^{\prime}}=\frac{\upmu }{{\mu }_{p}}$ and ${\theta }^{^{\prime}}=\frac{{T}_{r}-{T}_{p}}{T-Tp}$ for every data point in the dataset. μ′ always takes a value greater than 0 and reaches a maximum value of 1 at T = *T*_*p*_, i.e., 0 ≤ µ′ ≤ 1. We have obtained the universal plot using temperature-dependent mobility at different *V*_*g*_. The dimensionless quantities ${\mu }^{^{\prime}}= \frac{\upmu }{{\mu }_{p}}$ versus ${\theta }^{^{\prime}}=\frac{{T}_{r}-{T}_{p}}{T-Tp}$ curves collapse into a single curve for different *V*_*g*_, as seen for pentacene OFET data in Fig. 3a.

The obtained universal curve is well fitted by an exponential function ${\mu }^{^{\prime}}=a\text{exp}\left(\frac{b}{{\theta }^{^{\prime}}+c}\right)$ with $a\cong 1.04, b\cong -0.65,$ and $c\cong -0.13$ being the fitting constants, respectively. With simple dimensionless normalization parameters we find a comprehensive universality, where all the mobility curves at different gate voltages collapse into a single curve, exhibiting a general form across different organic semiconducting materials of the same hopping mechanism, as evidenced from Fig. 3b.

**Figure 4:**
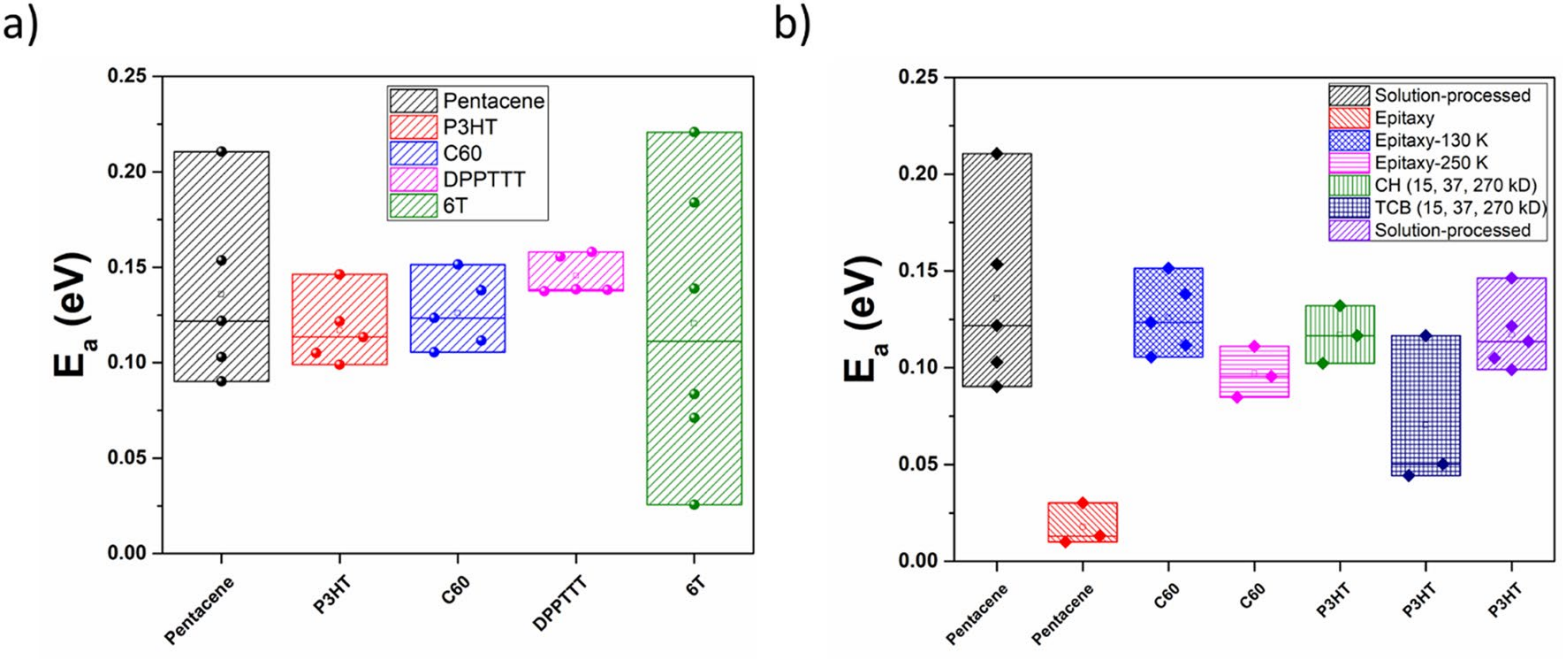
(**a**, **b**) Plots consisting of activation energy (E_a_) derived from the Renormalization Model, accessed by different OFETs. The horizontal axes denote the semiconductor for which the activation energy E_a_ is plotted on the vertical axes. For each organic system, the plots display the entire range of E_a_ values, with the data points being indicated by solid symbols. The minimum and maximum E_a_ values are marked by the topmost and bottommost data points, respectively. The corresponding mean and median E_a_ values for each OFET are indicated by the unfilled square and horizontal line, respectively, within each box; (**a**) activation energies (E_a_) accessed by each OFET as calculated from the range of gate voltages used in calculating the mobility-temperature dependence for each molecule. The E_a_ values have been calculated from the values of T_r_ and T_p_ for each curve and are shown for each OFET (filled spheres). The inset shows the semiconducting material whose E_a_ is plotted; (**b**) variation in the range of E_a_ accessed by each OFET fabricated using different processing techniques for the same semiconducting material. The inset represents the different processing conditions used as indicated by varying box patterns.

Furthermore, the knowledge of the *T*_*r*_ value enables us to calculate the value of the activation energy for that temperature-dependent mobility curve. The activation energy *E*_*a*_ can be expressed in terms of *T*_*p*_ and *T*_*r*_ as ${E}_{a}={k}_{B}\text{ln}\left(2\right)\frac{{T}_{r}{T}_{p}}{{T}_{p}-{T}_{r}}$, by evaluating the Bassler model equation at T = *T*_*p*_ and T = *T*_*r*_. Section S1 in the Supplementary Information gives a more detailed derivation of this relationship. Figure 4a shows the obtained/calculated range of activation energies, which are accessible to five different molecules by varying the gate voltage. In general, at low gating (low energy state filling) charge carriers require more energy for the hopping to occur, and this activation energy decreases as the gating voltage is increased (more negative values). The strength of this approach, in comparison to SR and the Bassler Model described earlier, is that a general rule to fix *T*_*r*_ as a function of *V*_*g*_, and the values of *E*_*a*_, are sufficient to capture the trends for five different organic systems. This, therefore, can be regarded as a generic fitting approach wherein the only descriptor required to convert the *µ*–*T* data to the *µ*′–*θ*′ data is the value of *T*_*r*_ for each *V*_*g*_ curve.

Figure 4b shows the activation energies accessible to each OFET that were fabricated under different processing conditions. Our results indicate that solution-processed P3HT films with chloroform (CH) require higher activation energies on average than the films grown in trichlorobenzene (TCB)^34^. The mobility is much higher for the TCB films as they require lower activation energy for the hopping transport. This is in line with previous reports on the solution processing of P3HT, wherein the higher mobility of TCB films is attributed to the lower volatility and improved crystallization resulting from deposition by spin-coating^35^.

Similarly, for C_60_, the films grown through hot wall epitaxy at *T*_*sub*_ = 250 °C show a lower band of activation energies accessed for almost similar values of gate voltage as compared to the films grown at *T*_*sub*_ = 130 °C^24^. This is again consistent with the overall level of crystallization of the resultant films, as evidenced by the AFM images and the value of the broadening of the DOS in either case (75.5 meV at *T*_*sub*_ = 130 °C and 53.5 meV at *T*_*sub*_ = 250 °C). A narrow broadening or a sharper DOS is seen in the case of molecules that have better crystallite ordering. Thus, the epitaxially grown pentacene films^36^ access a small range of activation energies and are less dispersed, as compared to the solution processed films^22^.

## Discussion

**Figure 5:**
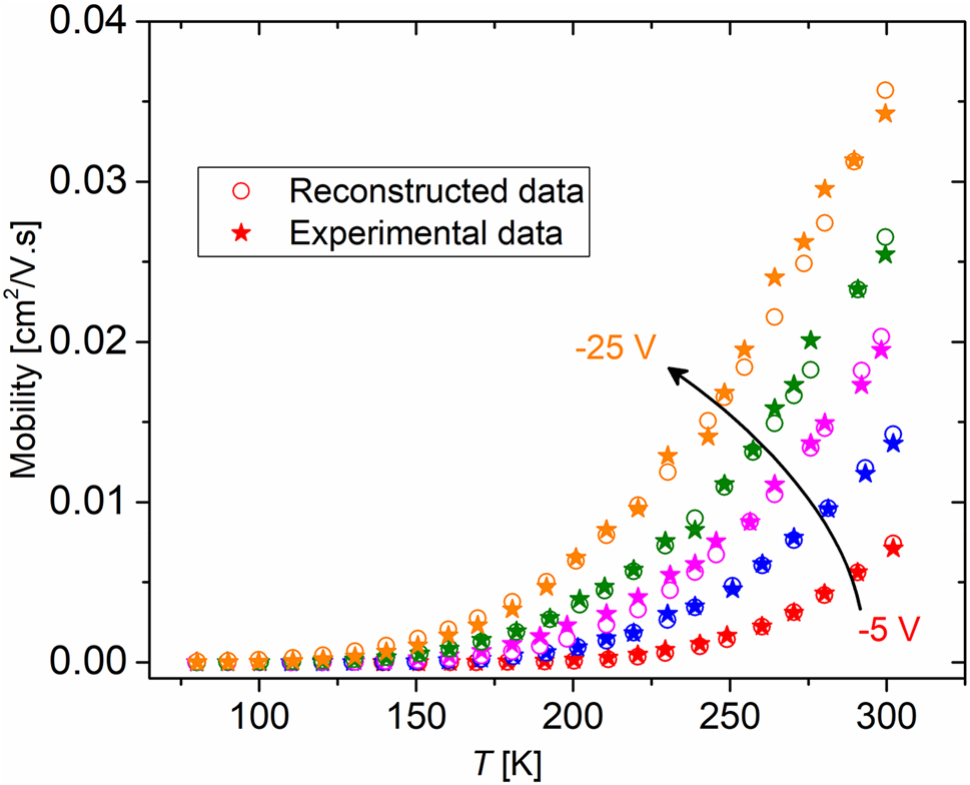
Comparison between the experimental data (blue symbols) and the reconstructed values from renormalization (red symbols) for pentacene OFET (data extracted from Ref.^19^).

We have shown previously that the activation energy *E*_*a*_ can be expressed as ${E}_{a}={k}_{B}\text{ln}\left(2\right)\frac{{T}_{r}{T}_{p}}{{T}_{p}-{T}_{r}}$. This relation is invertible for all molecules as both the original relation and the universal relation express mobility as an exponential function of the negative of the reciprocal temperature. The original and reconstructed fits for pentacene are shown in Fig. 5.

This approach is not only useful for studying the effect of gate voltage on the activation energy, but also that of the processing conditions such as the choice of solvent, polymer weight, and physical growth temperature, to name a few. This approach could also be extended to other techniques of controlling the extent of energy state filling such as chemical (or vacuum de-doping). Also, we show that the choice of *T*_*r*_ does not alter the nature of the function that fits the scaled data (only the coefficients *a, b, c* vary). We illustrate this in Section S2 of the SI with the help of temperature-dependent four-probe mobility data of a 2L-pentacene OFET^36^, where mobility at T = *T*_*r*_ was taken as 70% of *µ*_*p*_. Another salient feature is that the general fit shown in Fig. 3b can also act as a screening technique to determine which OFETs exhibit thermally activated hopping transport, with the help of very few measurements at different temperatures. For example, with mobility measurements at two different temperatures under constant gate voltage, with one at *T*_*p*_ and another below *T*_*p*_, the *µ*′ values of these points can be calculated as ${\mu }^{^{\prime}}= \frac{\mu }{{\mu }_{p}}$ and subsequently the corresponding *θ*′ values can be extracted from the plot (alternatively, the exponential equation) of the universal fit (Fig. 3b). From the *θ*′ values thus obtained, the *T*_*r*_ value of the data point below *T*_*p*_ can be determined. By virtue of the definition of the scaling relations, upon calculating *µ*′ and *θ*′ values at T = *T*_*r*_*,* we obtain values of 0.5 and 1 for *µ*′ and *θ*′, respectively, if the transport in the OFET occurs through thermally activated hopping. Hence, this not only serves as a screening/sanity check for the nature of charge transport but also helps in evaluating the activation energy at the applied gate voltage with the help of the *T*_*r*_ value determined thus.

A summary of the different data-fitting approaches is available in Table 2.

**Table 2:** A qualitative comparison of the three fitting approaches—Symbolic Regression, Bassler model and Renormalization model, as inferred from our results.

SR fitting	Accuracy and interpretability, but no generality. Considers non-linear terms
Bassler model	Physics-based fitting, but material- and range-specific. Not accurate beyond the bounds selected above, so not general
Renormalization fitting	General and interpretable approach but requires rescaling depending on the physics at play. Reconstruction is less accurate compared to Bassler and SR

## Conclusions

In this work, we use gate voltage and temperature-dependent OFET data from various small molecules/polymers and show that symbolic regression outperforms physics-inspired fitting techniques such as the Bassler model and the renormalization approach in fitting the OFET data, by one order of magnitude in the corresponding MAE values, although not offering generality achieved through physics-inspired fitting approaches. Through simple graphical value extraction, we demonstrate that the hopping mechanism in OFETs can be described by a general equation that relates normalized mobility with a scaled, dimensionless temperature descriptor. It is challenging to come up with a single model that accurately captures mobility characteristics in both low and high temperature regions; however, the SR outcome and physics-inspired model prove to be better than the baseline in this regard. Although powerful mathematical approaches like symbolic regression may offer reasonably good performance in terms of accuracy of fits, the physical interpretability is often incomplete and not consistent with the established know-how. We also proffer that a similar methodology can be extended to other transport mechanisms wherein the mobility is normalized with respect to the highest observed value and the temperature is then reduced to a dimensionless quantity that encompasses the dependency of the mobility on both the temperature and another experimental variables such as the gate effect or other processing conditions.

## Supplementary Information

Supplementary Information.
