# Supplementary material for: Comparing data driven and physics inspired models for hopping transport in organic field effect transistors
Source: Sci Rep. 2021 Dec 8;11:23621. doi: 10.1038/s41598-021-02737-7 (PMC8654921; doi:10.1038/s41598-021-02737-7)
Supplement: Supplementary file 1 — Supplementary Information. [file 41598_2021_2737_MOESM1_ESM.docx]

**Supplementary information for**

Comparing data driven and physics inspired models for hopping transport in organic field effect transistors

Madhavkrishnan Lakshminarayanan^1,2#^, Rajdeep Dutta^3#^, D V Maheswar Repaka^2^, Senthilnath Jayavelu^3*^, Wei Lin Leong^1*^, Kedar Hippalgaonkar^2,4*^

1. School of Electrical Electronic Engineering, Nanyang Technological University, 50 Nanyang Avenue, Singapore 639798
2. Institute of Materials Research & Engineering, Agency for Science, Technology and Research (A*STAR), Singapore 138632
3. Institute for Infocomm Research, Agency for Science, Technology and Research (A*STAR), Singapore 138632
4. School of Materials Science and Engineering, Nanyang Technological University, 50 Nanyang Avenue, Singapore 639798

# *correspondence to [j_senthilnath@i2r.a-star.edu.sg](mailto:j_senthilnath@i2r.a-star.edu.sg), [wlleong@ntu.edu.sg](mailto:wlleong@ntu.edu.sg), [kedar@ntu.edu.sg](mailto:kedar@ntu.edu.sg)

**Figure S1: Temperature-dependent mobility data for (a) P3HT and (b) C_60_ OFETs
[reproduced data from ref** ^1,2^**]**


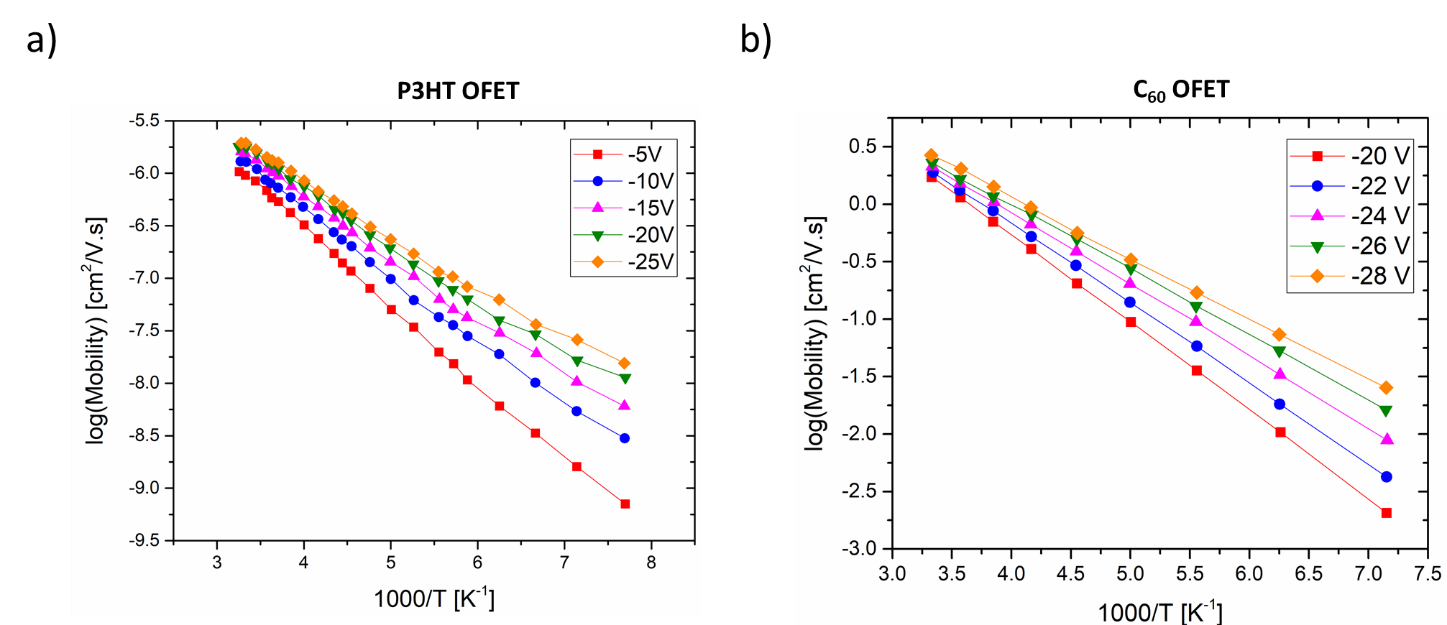


The logarithmic mobility of P3HT and C_60_ OFETs is plotted against the reciprocal temperature (in K^-1^) for different values of gate voltage (*V_g_*). The activation energy of each curve can be extracted from its slope as $E_{a}=\left| slope \right|*k_{B}*2303$, where k_B_ is the Boltzmann constant.

**Figure S2: SR mutation and crossover operations**


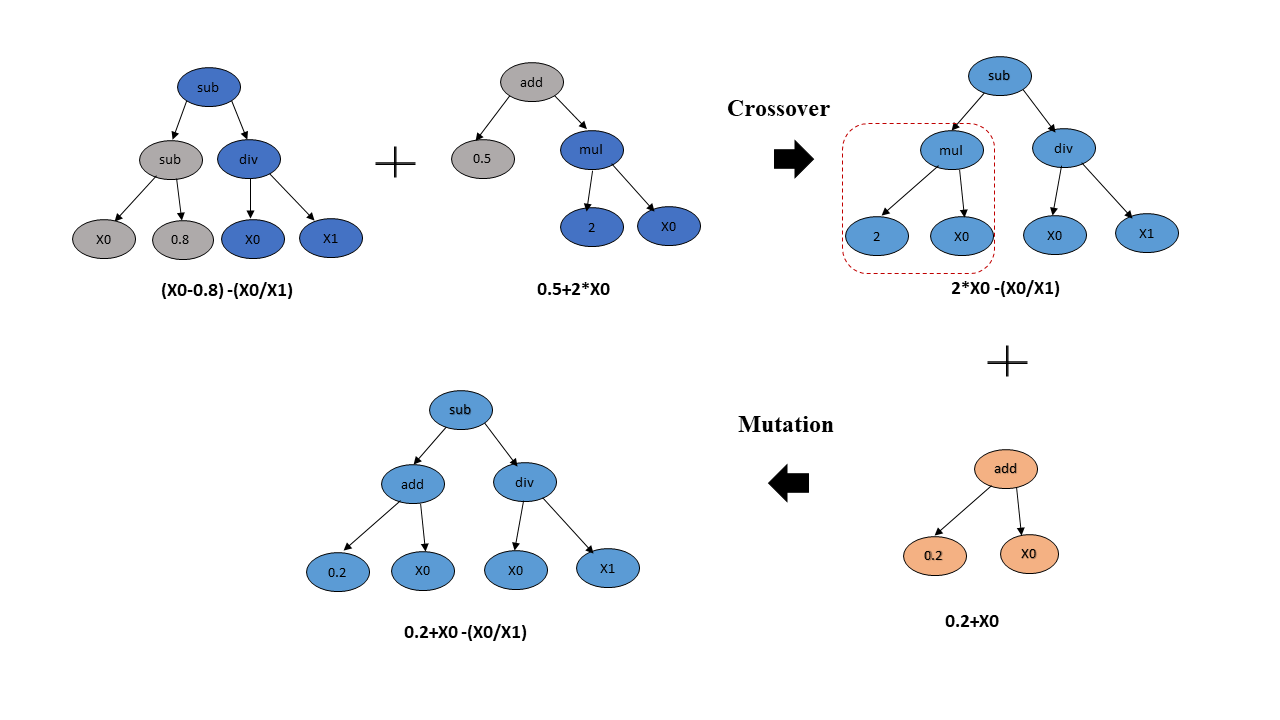

We provide here a precise mathematical overview of a generic Symbolic Regression (SR) problem. Consider a dataset (X,y) of *n* input variables and one output variable, where $X \epsilon\mathbb{R}^{n}, y \epsilon\mathbb{R}$. SR seeks to find a function $f\left( X \right) : \mathbb{R}^{n}\mathbb{\longrightarrow R}$that minimizes an error metric $E_{m}(y, f(X))$ between actual and predicted values. The optimal function can be represented as: $f^{*}=\arg\min_{f} E_{m}(y, f(X))$. Genetic Programming (GP) is an evolutionary algorithm used in functional optimization^3^, where candidate solutions are represented by computer-generated programs or trees made up of numbers, symbols, and functions^4^. GP candidates get updated at every generation (iteration) using *Tournament Selection* followed by *crossover* and *mutation*^3^ operations. GP applies crossover and mutation operators within the tree structures corresponding to candidate programs, as shown in Figure S2. The exploitation and exploration phases of the evolutionary search are considered by crossover and mutation operations, respectively, which we describe briefly in the following.

In the Tournament selection procedure, random individuals from a population are chosen to construct a pool to play a tournament amongst themselves. The tournament winner, i.e., the individual (tree) with the highest fitness, is carried forward to the next operation. The pool size needs to be carefully selected as a large pool size discourages weak individuals to participate in the tournament. Crossover propagates information from parents to offspring to improve the fitness of the next generation. A crossover operation in GP needs at least two tournaments to find out a parent and a donor. A crossover takes the winner of a tournament and selects a random subtree from it to be replaced by a donor determined by another tournament. Also, the donor has a randomly selected subtree that is inserted into the parent to form an offspring. Mutation enhances the exploration capability of the evolution process and maintains the diversity of a population by random replacement(s) of subtrees or nodes.

Some important tuning parameters associated with GP are population size, tournament size, crossover rate, mutation rate, tree depth bounds, and parsimony coefficient. The compactness of SR outcome expressions is determined by the related parsimony coefficient^3^.

**Tables T1-T3: Activation energy *E_a_* calculated from the slope of log(Mobility) versus 1/T plots compared with the *E_a_* extracted from the *T_r_* value of each *V_g_* curve as** $E_{a}=\boldsymbol{k}_{\boldsymbol{B}}\ln\left( \boldsymbol{2} \right)\frac{\boldsymbol{T}_{\boldsymbol{r}}\boldsymbol{T}_{\boldsymbol{p}}}{\boldsymbol{T}_{\boldsymbol{p}}\boldsymbol{-}\boldsymbol{T}_{\boldsymbol{r}}}$

The following tables compare the values of the activation energies as obtained from the slope of the temperature-dependent mobility curves (as shown in Figure S1) and those obtained from the *T_r_* value of each *V_g_* curve that was used for performing the renormalization.

*T1: C_60_ OFET*

| V_g_ (V) | E_a_ from slope (eV) | E_a_ from T_r_ (eV) |
| --- | --- | --- |
| -20 | 0.15144 | 0.1483 |
| -22 | 0.13795 | 0.1288 |
| -24 | 0.12342 | 0.1207 |
| -26 | 0.11166 | 0.1134 |
| -28 | 0.10556 | 0.1063 |

*T2: P3HT OFET*

| V_g_ (V) | E_a_ from slope (eV) | E_a_ from T_r_ (eV) |
| --- | --- | --- |
| -5 | 0.14626 | 0.1328 |
| -10 | 0.12158 | 0.1299 |
| -15 | 0.1135 | 0.1191 |
| -20 | 0.10503 | 0.1159 |
| -25 | 0.09904 | 0.1008 |

*T3: Pentacene OFET*

| V_g_ (V) | E_a_ from slope (eV) | E_a_ from T_r_ (eV) |
| --- | --- | --- |
| -5 | 0.21058 | 0.1891 |
| -10 | 0.15358 | 0.1345 |
| -15 | 0.12192 | 0.1172 |
| -20 | 0.10292 | 0.1014 |
| -25 | 0.09025 | 0.0882 |

**Figure S3: The hopping picture in a disordered polymeric semiconductor represented in terms of both energy and distance coordinates: the electronic Density of States (DOS) is characterized by the value of its energy broadening (σ). On the right, an illustrative single hopping event between two sites i and j, is shown, where sites i and j are characterized by electronic wavefunctions ψ_i_ and ψ_j_ respectively, and energies ε_i_ and ε_j_ respectively. Here, “a” represents the extent of wavefunction localization and “b” represents the distance between sites i and j.**


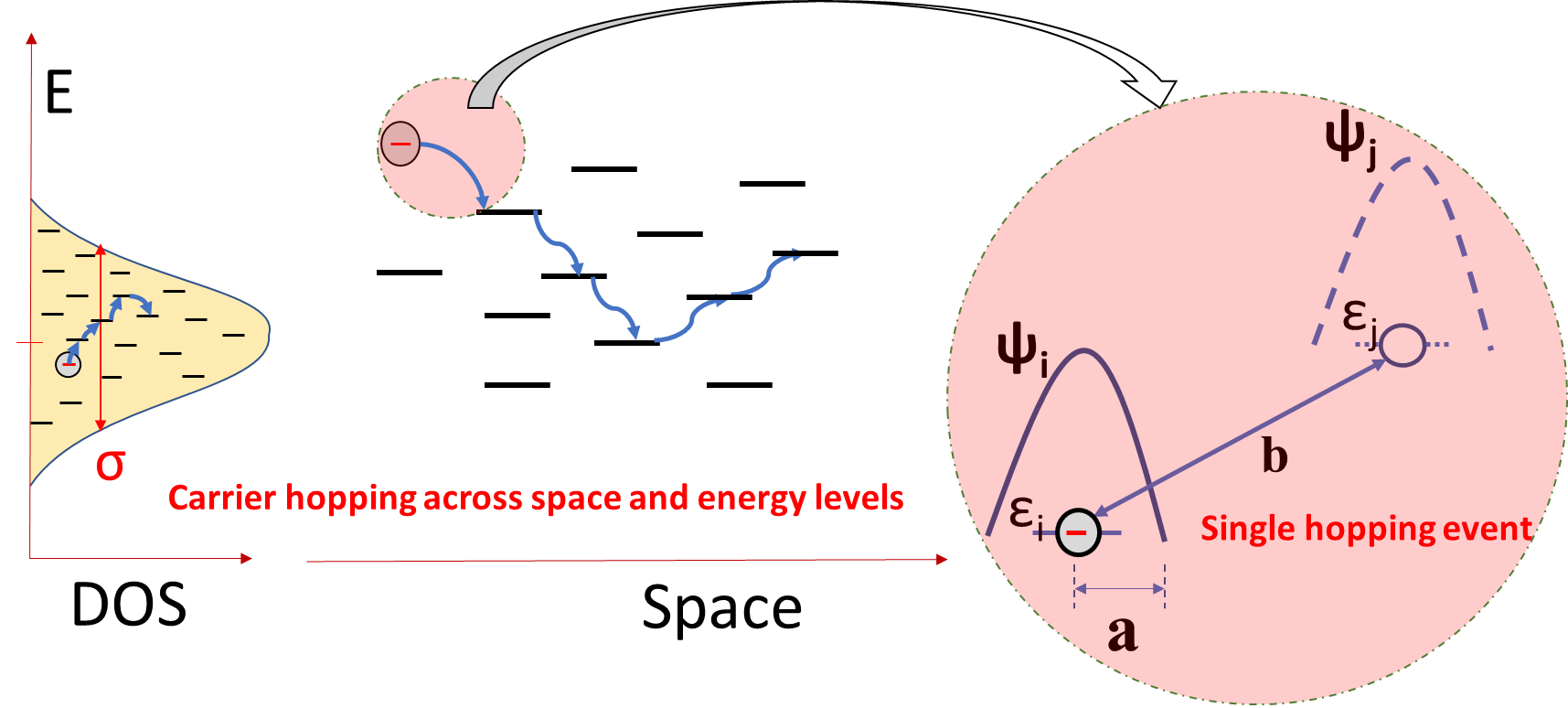


**Section S1: Derivation of activation energy *E_a_* in terms of *T_r_***

The field-effect mobility exhibits an Arrhenius relation with temperature, *i.e.*, $\mu\propto\exp\left( -\frac{E_{a}}{k_{B}T} \right)$. Upon expressing $\mu_{p}$ at ${T=T}_{p}$ and $\mu_{r}$ at ${T=T}_{r}$, their ratio can be expressed as $\frac{\mu_{p}}{\mu_{r}}=\frac{\exp\left( -\frac{E_{a}}{k_{B}T_{p}} \right)}{\exp\left( -\frac{E_{a}}{k_{B}T_{r}} \right)}$.

Since $\frac{\mu_{p}}{\mu_{r}}=2$ by our definition, the ratio can be rewritten as $2=\exp\left[ -\frac{E_{a}}{k_{B}}\left( \frac{1}{T_{p}}-\frac{1}{T_{r}} \right) \right]$.

Upon taking the natural logarithm on both sides, the activation energy can thus be expressed in the form $E_{a}=k_{B}\ln\left( 2 \right)\frac{T_{r}T_{p}}{T_{p}-T_{r}}$.

In our work, we fix a value of $T_{p}=300 K$ and hence the only additional parameter required to calculate $E_{a}$ as per the above equation is $T_{r}$. This is obtained from the temperature-dependent FET mobility curves as explained in the “Data description” section of the manuscript. Thus, *T_r_* is the characteristic temperature having different values for different semiconducting OFET system, as their activation energies vary. At the same time, it is important to note that this approach requires making choices of the scale invariant point beforehand.

**Section S2: Comparison of the renormalization for transport showing two different regimes and with different** $\frac{\mu_{r}}{\mu_{p}}$ **ratios**

In this Section, we illustrate that the renormalization approach works for (i) OFET data where two different transport regimes are observed, and (ii) when a different value of $\frac{\mu_{r}}{\mu_{p}}$ ratio is chosen (other than 0.5 chosen in the manuscript).

We have considered the temperature-dependent field-effect mobility data of a 2L-pentacene OFET, that was obtained through 4-probe measurement, published by Xinran Wang et al^5^. Since there is a visible transition in the behaviour of the mobility curves under different gate bias as shown in Figure S4, reproduced from the paper.

The dotted lines in Figure S4 separate both metallic and insulator regimes under the gate bias of -20 and -35 V.


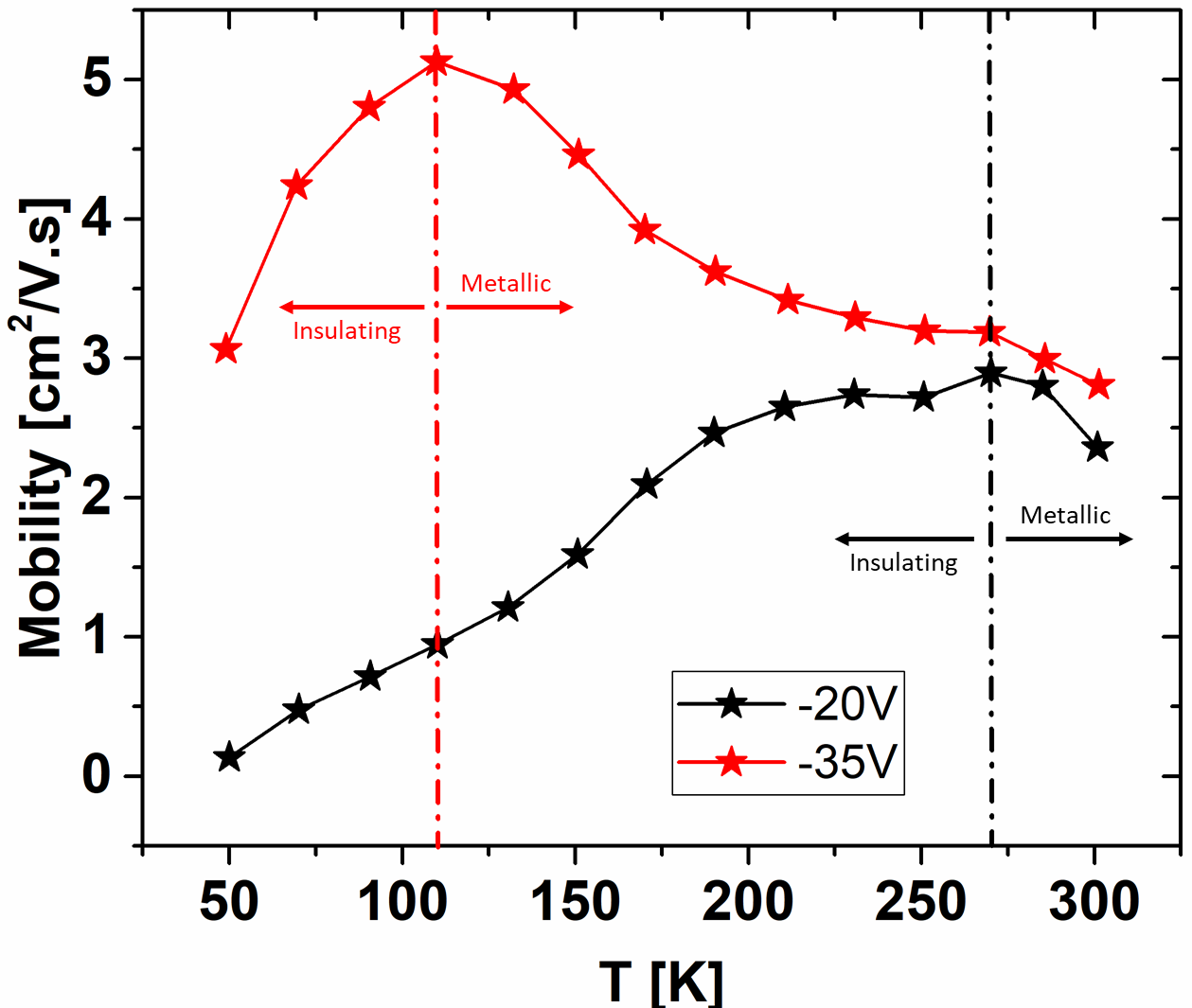


Figure S4: Temperature-dependent field-effect mobility data of 2L pentacene taken from Ref <^5^>. Dotted lines here indicate the shift from metallic to insulator behavior.

Since our generalizable model relies on Arrhenius transport, we restrict our analysis to the insulating regime, while the peak temperature allows us to separate the two transport regimes. We also note that the curve corresponding to -35 V does not have datapoints near half of the peak mobility, (~2.5 cm^2^/V.s). Hence, we have selected our *T_r_* in such a way that the mobility at *T_r_* is 70% of the value of the peak mobility. Upon performing the renormalization with these choices of *T_p_* and *T_r_* on the insulating regime, we find that the data follows an exponential relationship between the scaled, dimensionless *μ’* and *θ’*, like the curves reported in our manuscript (where $\frac{\mu_{r}}{\mu_{p}}=0.5$), *i.e.*, $\mu^{'}=a\exp\left( \frac{b}{\theta^{'}+c} \right)$ with $a\cong1.07, b\cong-0.28,$ and $c\cong-0.38$ being the fitting constants, respectively. The plot between the scaled values of *μ’* and *θ’* and the corresponding exponential curve that fits the scaled data (black dotted line) is shown in Figure S5.





Figure S5: Scaled mobility data of a 2L pentacene OFET under the insulating transport regime at two different gate voltages (coloured circles) for 2L pentacene OFET and the corresponding exponential curve that fits the scaled data (black dashed line) (data extracted from Ref <^5^>)

**References:**

1. Meijer, E. Ph.D. thesis: Charge transport in disordered organic field-effect transistors. (Technical University of Delft, 2003).

2. Ullah, M. *et al.* Dependence of Meyer-Neldel energy on energetic disorder in organic field effect transistors. *Appl. Phys. Lett.* **96**, 1–4 (2010).

3. GitHub - trevorstephens/gplearn at ad57cb18caafdb02cca861aea712f1bf3ed5016e. Available at: https://github.com/trevorstephens/gplearn/tree/ad57cb18caafdb02cca861aea712f1bf3ed5016e. (Accessed: 7th February 2021)

4. Sun, S., Ouyang, R., Zhang, B. & Zhang, T. Y. Data-driven discovery of formulas by symbolic regression. *MRS Bull.* **44**, 559–564 (2019).

5. Zhang, Y. *et al.* Probing Carrier Transport and Structure-Property Relationship of Highly Ordered Organic Semiconductors at the Two-Dimensional Limit. *Phys. Rev. Lett.* **116**, (2016).
